# Using a community-based system dynamics approach for understanding inclusion and wellbeing: a case study of special needs education in an eastern African refugee camp

**DOI:** 10.1186/s13031-021-00390-5

**Published:** 2021-07-23

**Authors:** Kelsey Werner, Gregory St. Arnold, Thomas M. Crea

**Affiliations:** 1 Boston College School of Social Work, 140 Commonwealth Avenue, Chestnut Hill, MA 02467 USA; 2 Jesuit Refugee Service, International Office, Borgo Santo Spirito 4, 00193 Rome, Italy

**Keywords:** Humanitarian setting, Special needs education, Inclusion, Community-based system dynamics, Complex problems, Causal loop diagram

## Abstract

**Background:**

Children with disabilities face unique challenges in humanitarian aid settings and education may provide protective measures against abuse and exploitation. There are growing calls for inclusive education of children with disabilities in formal education, but little guidance exists on how to enhance inclusion in complex and resource-constrained contexts of humanitarian settings.

**Case presentation:**

This study used a community-based system dynamics approach to understand key stakeholders’ perspectives of the drivers and effects of inclusion and wellbeing for children with disabilities, and to elicit recommendations to enhance educational inclusion in a refugee camp in Eastern Africa. Community-based system dynamics sessions, designed based on group model building scripts and facilitated by a team of four people, took place with organization staff, community leaders, and parents and caregivers of children with disabilities. The process produced a causal loop diagram depicting the stakeholders’ perspectives of how multiple components interact in a system to drive inclusion and wellbeing of children with disabilities over time.

**Conclusions:**

Findings indicate participants have a broad conceptualization of inclusion, highlighting the value of community interaction and importance of meeting basic needs, and also demonstrate that including children in mainstream educational settings in a complex humanitarian context requires a more nuanced approach given the lack of existing resources to support Western models of educational inclusion fully.

## Background

More than one billion people globally have a disability and more than 200 million experience significant difficulties in functioning [1]. According to the UN Convention on the Rights of Persons with Disabilities, “persons with disabilities include those who have long-term physical, mental, intellectual or sensory impairments which in interaction with various barriers may hinder their full and effective participation in society on an equal basis with others” [2]. An estimated 6.7 million persons with disabilities live in forcibly displaced situations worldwide [3]. Refugees with disabilities face unique challenges and often experience stigma and discrimination [4]. For children who are refugees, education can provide protection against abuse and exploitation [5]. Yet, little formal guidance exists in providing education to refugee children with disabilities, beyond a call for inclusive education that dismantles barriers to participating in education [6, 7]. The purposes of this study are to use Community Based System Dynamics (CBSD) to explore key stakeholders’ perspectives of the complex drivers and impacts of inclusion and wellbeing of children with disabilities in a refugee camp, and to identify places to intervene to promote inclusion.

Children and adolescents with disabilities face significant stigma in refugee camps [3]. These attitudes pose barriers to accessing education, interacting with peers, and can also place children at increased risk of gender-based violence: physical violence for boys, and sexual violence for girls [3]. Given these protection concerns, specialized services for children with disabilities, including separate schools, ensure children’s needs are being met [3]. Individual case management has been found to be a successful protective factor for persons with disabilities as it can help provide psychosocial support and increase access to educational supports through advocacy [3]. While Western models of educational inclusion focus on mainstreaming children with disabilities [7], little practical guidance currently exists on how best to serve the needs of students with disabilities in a refugee camp.

A prevailing strategy is to include children with disabilities in mainstream classrooms [7]. Mainstreaming in the US has been linked to improved school enrollment and retention of children with disabilities [8] as well as improved academic outcomes for students without disabilities [9]. Yet, limited research exists on disabilities education for children who are refugees, and in particular are living in refugee camps. In these contexts, where class sizes can exceed 60–100 students per teacher, mainstreaming students with disabilities is not likely to result in their receiving needed academic and other supports in the classroom [10, 11]. Little research currently exists to suggest whether it is preferable to serve children with disabilities separately in their own classrooms, or together in mainstream classrooms alongside students without disabilities. The Women’s Refugee Commission found that teasing and emotional abuse faced by children with disabilities was worse in situations where children with disabilities are served in segregated classrooms, often deterring students from attending school [3]. By contrast, Reilly found low attendance and high dropout rates for children with disabilities attending mainstream classrooms [12].

Limited research available suggests a number of barriers to special needs education and mainstreaming in refugee camps. Further complicating attempts to support inclusive education is a lack of understanding of how these barriers interact with one another in the complex context of the refugee camp. Refugee camps have been characterized as complex systems where international, national, organizational, and human factors interact in dynamic ways [13]. Further, camps are subject to shocks or unpredictable events that impact the function of the system. Inadequate understanding of how the system functions jeopardizes interventions and programs designed to support refugees [14]. In addition to the complexity of the camp context, other research highlights the complexity of mental health service provision [15], creating equity and strengthening inclusion in education for children with disabilities [16, 17]. In addition to these layers of complexity, the conceptual approaches to disability from the medical model, framing disability as an individual condition that must be repaired or normalized, to the social model, situating disability as a product of social structures, and a variety of other approaches, shape the interpretation and operationalization of inclusive education and mainstreaming in any context [18]. Some research seeks to understand the perspective of key stakeholders such as caregivers, teachers, disability advocates, and people with disabilities without prescribing an assumed understanding or conceptual approach to disability. Exploring different perspectives and how they align with or differ from existing conceptual approaches helps deepen understanding of experiences in real world contexts [19–21]. More research is needed to understand the layers of complexity of inclusive education of children with disabilities from the unique perspective of stakeholders in refugee camp contexts.

System dynamics is one approach to understanding how factors interact in complex systems. System dynamics uses qualitative causal maps and formal quantitative models to explore how factors change over time from the endogenous, feedback perspective [22]. In the field of system dynamics, CBSD uses participatory group model building to engage stakeholders embedded in the system in developing system dynamics models [23]. CBSD places an emphasis on building capabilities of stakeholders to understand how complex systems change over time from a feedback perspective [23]. The method provides tools to visually depict the groups’ perspective of complex systems.

Causal loop diagrams, one type of qualitative causal map, helps visualize components in a system and their positive or negative relationships with one another using arrows and feedback loops. The process of collaboratively and iteratively developing causal loop diagrams helps build consensus on how the system is structured and identify places to intervene [24–26]. In the current study, CBSD was used to convene key stakeholders to develop a causal loop diagram of the drivers and effects of inclusion and wellbeing for children with disabilities and elicit recommendations to enhance inclusion in a large refugee camp in East Africa.

## Case presentation

### Setting

Specific details about the setting have been excluded to protect the identity of this vulnerable population. The refugee camp is located in East Africa in the poorest county in the host country. Over half of the refugees fall below the international poverty line for extreme poverty (US$1.90 per capita per day), which is higher than the national rate of the host country [27]. The refugee camp has evolved as a place where refugees and members of the local community can live in close proximity and access and benefit from services and support offered as a result of the refugee presence. The population continues to grow, with over half of the current population having arrived in the past 5 years and totaled over 200,000 as of April 2021 [28]. Refugees and asylum seekers originate from nine countries including South Sudan, Sudan, Somalia, Ethiopia, Rwanda, Eritrea, Burundi, Uganda, and Democratic Republic of the Congo [28]. Over half of the refugee population in the camp are children under age 17, with around 30% of children under 11 years of age [28].

Since 2010, Jesuit Refugee Service (JRS) has operated Mental Health Centers in the refugee camp to provide support for people with disabilities. There are a wide range of disabilities served, including physical, mental, emotional, and learning differences and disabilities. Services are open to all children with disabilities, regardless the type of disability or level of functioning. In 2016, these centers transitioned to Special Needs Centers with an expanded focus beyond protection to include education, training, and support. Children served by the Special Needs Centers now receive much more educational supports in the form of receiving functional assessments, Individualized Education Plans, and increased resources such as textbooks.

In spite of these advances, children with disabilities are typically not included in mainstream school activities. Currently, approximately 100 children are served in six of the 26 schools in the refugee camp in separate special education units. Thus, the vast majority of children with disabilities in the refugee camp have limited or no contact with children in mainstream educational settings.

### Project design

CBSD sessions were completed over the course of 2 weeks. All sessions were facilitated by a team of four people, including a researcher and three JRS local staff. The three JRS local staff participated in a daylong training on CBSD prior to the start of sessions. CBSD uses group model building scripts, or structured activities, as the building blocks for the modeling process [23, 29]. CBSD sessions used the following scripts: Presenting the Reference Mode, Variable Elicitation, Initiating and Elaborating a Causal Loop Diagram, Model Review, Action Ideas, and Dots (see Table 1). Scripts were tailored to address inclusion and wellbeing and adapted for language and literacy considerations. The adapted scripts were compiled in a facilitation manual and represented in a pictorial facilitation manual for training purposes.

**Table 1:** Group Model Building Scripts

Script Name	Description	Output
Negotiating a reference mode	Participants negotiated how to represent historical trends of inclusion and wellbeing of children with special needs	Graphs depicting changes over time and hoped and feared trajectory for the future
Variable Elicitation	Participants named factors or variables that impact or are impacted by the changes in inclusion and wellbeing over time depicted in the previous activity	List of factors for the causal loop diagram from each stakeholder group
Initiating and Elaborating a Causal Loop Diagram	Facilitators led participants through describing and drawing the causal connections and feedback mechanisms between variables listed in the previous activity	1 Causal loop diagram per stakeholder group
Model Review	Facilitators presented the combined causal loop diagram capturing diverse stakeholder groups perspectives and added or refined model structure based on participant feedback	1 refined causal loop diagram per center
Action Ideas	Participants generated ideas of places to intervene to enhance inclusion and facilitators captured the ideas in lists and by mapping the ideas on to the causal loop diagram based on where participants thought they impacted the system	List of action ideas of places to intervene in the system from each center
Dot Vote	Participants used dot stickers to vote for the ideas they felt were most important to prioritize	Prioritized list of potential actions from each center

**Fig. 1:**
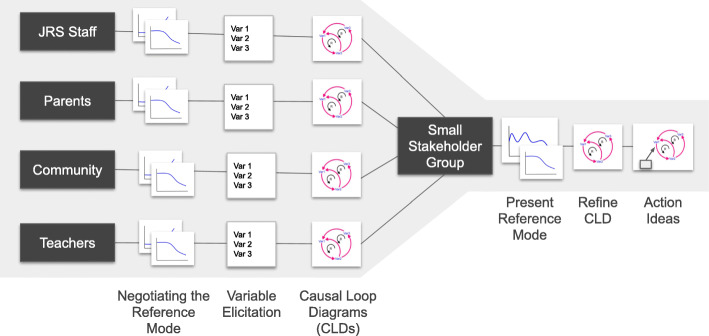
Process Overview: Overview of the project design including stakeholder groups, group model building scripts and outputs. This process was replicated in two Special Needs Centers

The following stakeholder groups were convened: (a) JRS national staff; (b) parents/families/caregivers of children with disabilities; (c) JRS Special Needs Center staff; and (d) community leaders. Recruitment was led by a local research manager and inclusion criteria varied by stakeholder group. Any JRS national staff were welcome to participate. Stakeholder groups b, c, and d were recruited from two JRS Special Needs Centers included in this study. Caregivers had to have a child enrolled in the JRS Special Needs Program at one of the two centers. Caregiver participation was not restricted by the type or severity of the child’s disabilities. JRS Special Needs Center staff participation was limited to staff at the two centers and community leaders were recruited from the community surrounding each center. Each stakeholder group included 8–12 people and was convened separately to understand their unique perspective of the problem. The languages used for facilitation varied across sessions, depending on the stakeholder group, and included English, Swahili, and Arabic. All outputs were translated into English by the facilitation team after each stakeholder session. Between one and three representatives from each stakeholder group were then convened jointly to build consensus around the system driving inclusion and wellbeing and generate recommendations for the future. This process was replicated with stakeholder groups b, c, and d at two JRS Special Needs Centers. See Fig. 1 for an overview of the group model building activities completed with each stakeholder group.

### Analysis

Causal maps were synthesized and refined through an iterative process by the research team and participants. After the session, causal loop diagrams from each stakeholder group were integrated into a combined causal loop diagram for each center by the research team. The combined causal loop diagram was refined by participants in the small stakeholder group session at each center and used to elicit action ideas. The resulting two refined causal loop diagrams were synthesized into one causal loop diagram.

## Results

**Fig. 2:**
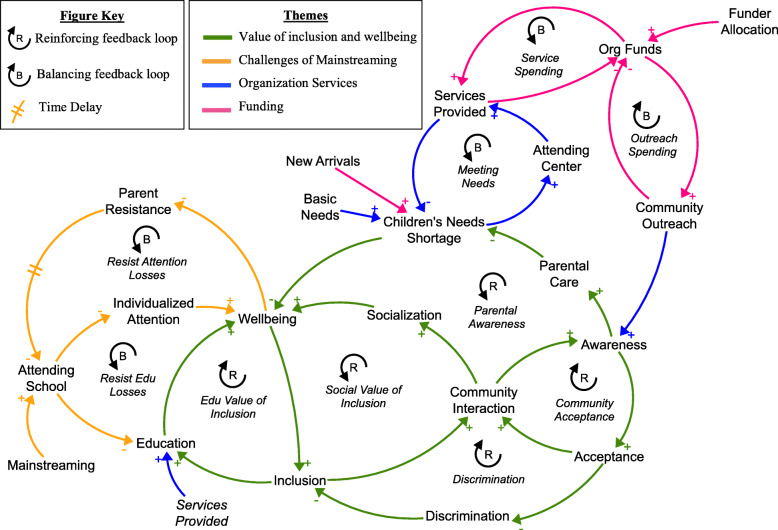
Synthesized Causal Loop Diagram of Inclusion and Wellbeing: Arrows with positive polarity (+) indicate an increase in the cause variable leads to an increase in the receiving variable or a decrease in the cause variable leads to a decrease in the receiving variable. Arrows with negative polarity (−) indicate an increase in the cause variable leads to a decrease in the receiving variable or a decrease in the cause variable leads to an increase in the receiving variable

The group model building activities elicited the following from seven different stakeholder groups: (a) two negotiated reference modes of how and inclusion and wellbeing of children with disabilities changed over time and their hoped and feared trajectories; (b) variables that impact or are impacted by inclusion and wellbeing of children with disabilities; and (c) a causal map of interconnections between variables. Each center produced refined causal loop diagrams and prioritized recommendations. Figure 2 captures stakeholder perspectives in the synthesized, high level causal loop diagram that includes key feedback loops and themes highlighted by different colors. There are two kinds of feedback loops, reinforcing and balancing. Reinforcing feedback loops amplify changes over time. Creating so called ‘virtuous’ growth or ‘vicious’ decline cycles, an increase leads to more of an increase or a decrease leads to more of a decrease. For example, an initial increase in community interaction leads to more awareness and greater acceptance, resulting in even more community interaction. Balancing feedback loops dampen or limit changes over time. In this balancing cycle, an increase feeds back around to a decrease, or a decrease feeds back around to an increase [30, 31]. For example, as organization funds increase more services are provided, depleting funds and limiting the services provided. The push and pull of feedback loops interacting in the system act as an initial dynamic understanding of changes in inclusion and wellbeing of children with disabilities over time. The feedback loops are named to describe their role in the system. Themes in the causal loop diagram include: (a) Value of inclusion and wellbeing (green arrows); (b) Challenges of mainstreaming (yellow arrows); (c) Organization Services (blue arrows); and (d) Funding (pink arrows). The following sections describe each theme in detail including the reinforcing and balancing feedback loops related to each theme.

### Value of inclusion and wellbeing

Participants described a variety of linkages where inclusion of children with disabilities and wellbeing reinforce one another. This theme is depicted in green arrows in Fig. 2. “Social Value of Inclusion” is a reinforcing feedback loop that describes how inclusion increases community interaction leading to more socialization and greater wellbeing. Participants described when a child is included in play they are more active and happier leading to greater physical and social wellbeing. Enhanced child wellbeing enables them to be included more. Greater community interaction also leads to more awareness and acceptance of children with disabilities, reinforcing community interaction as seen in the loop “Community Acceptance”. Community acceptance also helps reduce discrimination, reinforcing inclusion shown in “Fighting Discrimination”. These dynamics can also create vicious cycles where children with disabilities are included less or excluded leading to less community interaction, lower awareness and less acceptance. This dynamic can reinforce discrimination and low inclusion.

“Educational Value of Inclusion” is a reinforcing feedback loop that describes how participants viewed education as a form of inclusion. Participants described how educational opportunities create greater independence for children with disabilities, enhancing their wellbeing and allowing them to be further included. Participants also described that children needed to have their basic needs met to ensure a minimum level of wellbeing in order for the child to be included and care from parents or caregivers is one way those needs are met. Participants discussed how awareness about disabilities helps parents understand their child’s needs and how to provide care. As parents become more aware, they provide more care to meet children’s needs, enhancing their wellbeing and enabling inclusion. This inclusion helps continue to spread awareness in the community which helps to reach more parents with children with disabilities, reinforcing care, wellbeing and inclusion as seen in “Parental Awareness”. Some participants also began to unpack inclusion as a construct such as specifying that it is a child’s right and includes access to services and community protection.

### Challenges of mainstreaming

There was significant discussion around mainstreaming and the connection between mainstreaming and inclusion. The synthesized model reflects the groups’ consensus, combining differing perspectives primarily between organization staff and caregivers. Mainstreaming was not seen as a direct promoter of inclusion or wellbeing. Mainstream classrooms in the refugee camp context often do not have the capacity to meet the educational needs of children with disabilities or provide individualized care. Participants described children receiving less individualized care and educational opportunities in mainstream classrooms than through services provided at the organization centers. This situation leads parents to resist their students attending schools to avoid potential losses that could harm their child’s wellbeing, depicted in the balancing loops “Resist Attention Losses” and “Resist Education Losses”. As children’s wellbeing is restored, parents and caregivers may encourage children to attend schools again creating fluctuations in attendance. Participants expanded on some of the positive aspects of mainstreaming, including greater socialization and other students gaining a greater understanding of children with disabilities. However, they indicated for mainstreaming to be successful it required greater accessibility in classrooms and additional specialized teachers and training.

### Organization services

Participants described the importance of service provision for inclusion and wellbeing. “Meeting Needs” illustrates how services are provided at the organization’s centers to children with disabilities to meet their basic needs such as providing medicine, nutrition, and education. Parents and caregivers identified poverty as a key barrier for meeting their child’s basic needs and therefore described services provided by the organization as crucial for wellbeing. “Meeting Needs” also highlights that some children may attend the center less if their needs are being adequately met, which could limit their access to other services provided at the centers such as education. Participants identified education as an important service that helps create greater independence, enhancing wellbeing and helping to prevent children from falling into poverty in the future. The organization also does community outreach to help spread awareness of the services they provide and promote acceptance of children with disabilities. Some participants described children receiving very little care prior to initial outreach. Outreach efforts can help jump start the cycles of community acceptance and parental awareness previously described. Participants elaborated the impact of community outreach as promoting child rights and acting as a barrier to stigma.

### Funding

The services provided by the organization are finite and dependent on outsides funds for provision. Organization activities including direct service provision and community outreach depletes funds available as seen in “Service Spending” and “Outreach Spending”. Participants described funding as primarily responsive to donors, not demand for organization services. This implies funder allocation is not closely responsive to different demands in the system, which means shocks to the system can leave communities vulnerable to unmet needs. Shocks to the system like an influx of new arrivals to the camp leading to increased demand for services or budget cuts reducing service provision can leave the organization unable to meet children’s needs. Participants described efforts to mitigate this effect through referrals to other organizations to meet needs. However, this process is also not coordinated to be responsive to demand, leading to some scenarios where a user may be referred to an organization that cannot provide the service. Participants also described some of the social effects of new arrivals and its impact on inclusion. New arrivals bring greater cultural diversity which they described as leading to more discrimination and rejection of children with disabilities. However, after some time living in proximity, new arrivals begin to integrate and interact more leading to harmony between groups that help prevent mistreatment of children. Participants indicated that the organization played a role in this process by promoting awareness through community outreach which encouraged communities to protect children.

### Participant recommendations

Based on insights from the causal maps, participants generated a set of ideas or recommendations of how to improve inclusion and wellbeing of children with disabilities. Recommendations were mapped on to the refined causal loop diagram based on where participants thought the idea impacted the system. Given the interconnectedness of the system and feedback effects, interventions may lead to greater inclusion and wellbeing through a variety of pathways rather than directly impacting inclusion. Participants suggested a number of ideas for expanding the type of services and access to services offered at the organization including medical specialist checkups, enhanced mental health services, extending hours, and increasing the number of centers to reach more children. Several ideas were aimed at supporting parental care including support groups, providing basic supplies like school kits or assistive devices, and promoting caregivers’ financial stability through job training or economic support. Participants also generated ideas to increase community interaction such as community-wide trainings on acceptance and protection and creating sports or music competitions open to all children. Other ideas aimed to achieve the socialization and education benefits of mainstreaming such as providing additional funds for education in centers, hiring more special needs teachers in mainstream schools, and providing scholarships for special education schools outside of the camp context.

## Discussion

The literature is largely silent on how to address educational inclusion for children with disabilities in humanitarian settings such as a refugee camp. In this study, engaging stakeholders in the process of group model building allowed for a variety of insights into the dynamic complexity of drivers and impacts of inclusion and wellbeing of children with disabilities. The model depicts the multiple pathways through which inclusion and wellbeing reinforce one another. The research captured multiple stakeholder groups’ perspectives of how inclusion is conceived more broadly, beyond a narrow educational perspective. At the time of the study, participants did not conceive of inclusion as the commonly held understanding of inclusion as mainstreaming in educational settings [8] because of the limits of mainstream classrooms in the refugee camp, but they acknowledged the theoretical benefits if conditions improved. Inclusion was also elaborated as a construct that includes inclusion in educational opportunities but also community acceptance and interaction. The reinforcing nature of inclusion and community acceptance and interaction was identified as a mechanism that helps fight discrimination. This dynamic highlights the need to strive for multiple forms of inclusion in refugee contexts, including in educational settings and the community.

The findings corroborate some existing evidence on mainstreaming, and extend it by capturing the dynamics unique to refugee camp contexts. Participants described the hypothetical benefits of mainstreaming including greater educational opportunities and increased socialization, but identified challenges specific to the refugee camp context. Integrating children with disabilities into mainstream classrooms that are not equipped to meet the individualized needs of children could result in lower child wellbeing. This finding aligns with existing literature highlighting the profound infrastructure and teacher capacity constraints in refugee camp contexts [3, 10, 11]. The model also highlights resistance from parents that could lead to oscillating periods of lower attendance rates, extending existing literature on high dropout rates [12] and the importance of the attitudes of children’s parents [32]. These findings indicate the need for a more nuanced approach to including children in educational settings in refugee camps to ensure the theoretical benefits of mainstreaming can be realized.

Outcomes from this study also demonstrate the value of meeting basic needs to enable conditions for inclusion. Services provided at centers help meet the basic needs of children which participants described as ensuring children were well enough to be included. Literature suggests services to children with disabilities should include psychosocial and educational support [3]. This study’s findings indicate that in the unique context of refugee camps, the endemic poverty and lack of opportunities for stable income may create challenges for caregivers to meet children’s basic needs. Services for children in refugee camps, therefore, may need to be expanded to meet basic needs, in addition to providing psychosocial and educational support to create the enabling conditions for inclusion. Yet, parents’ accessing services solely to meet minimum basic needs may hinder other supports for children. For example, if children only attend centers for periods of time when parents are unable to provide adequate care or for a few hours to receive certain services, their access to other psychosocial and educational services may be limited.

The findings highlight some unique exogenous factors, i.e., factors external to the system, such as funding. Participants described organizational funding as not being closely aligned with demands for services, leaving service provision vulnerable to shocks to the system like a sudden influx of refugees or budget cuts. This lack of alignment has implications for inclusion of children with disabilities. If children’s basic needs are not being adequately met through service provision, the resulting decrease in wellbeing could trigger vicious cycles of less community interaction and acceptance, and more discrimination.

The recommendations generated by participants highlight the importance of understanding the unique complexity of refugee camps. Participants articulated ideas to enhance inclusion through a variety of pathways that may not seem to directly impact inclusion if ignoring the complexity of the context, such as supporting caregiver financial stability or enhancing community interaction. Engaging a variety of stakeholders, both organization staff and caregivers, helped create consensus around a more nuanced view of inclusion and participation in the research process helped for rapid translation of insights into practice. JRS is embarking on a pilot to provide services and educational opportunities to children of all abilities in an Inclusive Education Center. This pilot essentially flips the concept of mainstreaming, tailored to the local context by including children without disabilities in classrooms with children with disabilities. Children with disabilities will still receive the individualized support they need while gaining the educational opportunities and socialization typically associated with interacting with other children in a mainstream classroom.

## Study limitations and areas for future research

This study has some limitations. The process of engaging stakeholders in modeling helped question previous assumptions about the value of mainstreaming for inclusion in refugee camps and point the way towards unique innovations to serve the needs of all children. However because of the high degree of engagement, the resulting model reflects the perspective of stakeholders with limited generalizability. This work was limited to the perspectives of certain stakeholder groups in two communities in one refugee camp. Additional CBSD sessions could be held to share back the model to validate the insights, as well as convene additional stakeholders such as children with disabilities, to understand a variety of perspectives on inclusion and explore potential contextual differences in other refugee camps. More work is needed to understand how different communities interpret inclusion, the value of inclusion and its connection to child wellbeing, and how these interactions may differ from the perspectives of a variety of organizations who intervene in this space.

The causal loop diagram provides initial insights into the complex and dynamic nature of inclusion and wellbeing. However, a simulation model needs to be developed to test if the model structure reproduces expected changes in behavior over time and to explore the impact of participant recommendations. Future research could include developing a formal simulation model from the causal loop diagram to gain deeper system insights such as simulating how this system generates changes in inclusion and wellbeing and identifying leverage points.

Iterative synthesis of the causal loop diagram may have been influenced by translation and the perspective of the researcher. Future studies could include modeling in additional languages and additional testing and refining of the model with key stakeholders to reduce researcher bias. Further, developing the capabilities of organization staff, refugees and members of the community themselves, is a first step toward building capacity to use the CBSD approach and empowering communities to understand not only inclusion, but a variety of complex social problems embedded in refugee camps. Additional use of CBSD will further develop capabilities, enabling a deeper understanding of inclusion and possible exploration of other problems.

## Conclusion

Findings from this work help inform an expanded, more nuanced view of the meaning of inclusion and reinterpret the value of mainstreaming as an inclusive education strategy given the constraints and unique complexity of a refugee camp. Inclusion enhances wellbeing but children must have their basic needs met to create the enabling conditions for inclusion. Therefore, organizations who provide a variety of services beyond education to children in refugee camps play a vital role in supporting inclusion. Funding to such organizations was not closely responsive to demands for services, leaving organizations susceptible to shocks and possibly unable to support children with disabilities.

Participants’ involvement in the CBSD process helped enable rapid translation of research into practice, resulting in an innovative inclusive education pilot. This approach holds promise for engaging a variety of stakeholders to understand the complex interactions between inclusion and wellbeing of children with disabilities in refugee camp contexts and build consensus to inform program design. More research is needed to gain deeper insights of where to intervene in this context, understand the unique perspectives of inclusion in other communities, and explore differences across refugee camps.

## Data Availability

Data sharing is not applicable to this article as no datasets were generated or analyzed during the current study.

## Abbreviations

CBSD: Community Based System Dynamics
JRS: Jesuit Refugee Service
